# Microbiological Non-Culture-Based Methods for Diagnosing Invasive Pulmonary Aspergillosis in ICU Patients

**DOI:** 10.3390/diagnostics13162718

**Published:** 2023-08-21

**Authors:** Ulrike Scharmann, Hedda Luise Verhasselt, Lisa Kirchhoff, Dan-Tiberiu Furnica, Joerg Steinmann, Peter-Michael Rath

**Affiliations:** 1 Institute of Medical Microbiology, University Hospital Essen, University of Duisburg-Essen, 45122 Essen, Germanyjoerg.steinmann@klinikum-nuernberg.de (J.S.);; 2 Institute of Clinical Hygiene, Medical Microbiology and Infectiology, Klinikum Nürnberg, Paracelsus Medical University, 90419 Nuremberg, Germany

**Keywords:** *Aspergillus*, aspergillosis, fungal infections, ICU, galactomannan, (1→3)-β-d-glucan

## Abstract

The diagnosis of invasive pulmonary aspergillosis (IPA) in intensive care unit (ICU) patients is crucial since most clinical signs are not specific to invasive fungal infections. To detect an IPA, different criteria should be considered. Next to host factors and radiological signs, microbiological criteria should be fulfilled. For microbiological diagnostics, different methods are available. Next to the conventional culture-based approaches like staining and culture, non-culture-based methods can increase sensitivity and improve time-to-result. Besides fungal biomarkers, like galactomannan and (1→3)-β-D-glucan as nonspecific tools, molecular-based methods can also offer detection of resistance determinants. The detection of novel biomarkers or targets is promising. In this review, we evaluate and discuss the value of non-culture-based microbiological methods (galactomannan, (1→3)-β-D-glucan, *Aspergillus* PCR, new biomarker/targets) for diagnosing IPA in ICU patients.

## 1. Introduction

Invasive pulmonary aspergillosis (IPA) is most common in patients with hematological malignancies, like acute myeloid and lymphoid leukemia (AML, ALL). But other immunosuppressive diseases or distinct circumstances, like a pulmonary virus infection or a long stay at the intensive care unit (ICU), are risk factors for an IPA as well [1,2,3,4,5]. In a multicenter study, a multivariate analysis to determine the risk of death was performed. Of 152 patients with invasive fungal infections, 92 (60.5%) died [6]. The diagnostic of IPA is crucial due to various criteria, which are non-fungal-specific. The infectious diseases group of the European Organization for Research and Treatment of Cancer and the Mycoses Study Group (EORTC/MSG) defined three classes for the IPA (proven, probable and possible), depending on which consensus criteria are fulfilled [7]. These criteria include host factors of the patients (e.g., previous illnesses or immunosuppressive therapy), clinical features (radiological signs, abnormalities in typical anatomical sides) and mycological evidence (culture or microscopical detection of fungal structures in respiratory material and detection of biomarkers, like galactomannan (GM) antigen or (1→3)-β-D-glucan (BDG) and DNA detection) [7]. As mentioned by Bassetti, patients in ICUs often do not have risk factors like hemato-oncologic patients. In these patients, risk factors for aspergillosis are treatment with corticosteroids in high doses, diabetes, chronic liver or pulmonary diseases (COPD), or malnutrition. Furthermore, influenza and other viral infections like COVID-19 are risk factors [8]. Typical radiological signs of aspergillosis are usually not found in ICU patients. For diagnosing invasive fungal diseases (IFD) in critically ill patients, Blot et al. defined criteria to discriminate between colonization with *Aspergillus* in the respiratory tract from an IPA. Here, only two categorizations are defined, the proven IPA (same as EORTC/MSG criteria) and the putative IPA [9]. Additionally, Schauwvlieghe et al. also defined a clinical algorithm to diagnose IPA in critically ill patients and Koehler et al. in patients with COVID-19 [3,4]. Finally, Bassetti et al. [8] defined a probable invasive aspergillosis in patients at risk (glucocorticoid treatment with prednisone equivalent of 20 mg or more per day, or ≤500 neutrophil cells/mm^3^, or COPD, or treatment with immunosuppressants, or hemato-oncological diseases, or organ transplantation, or HIV-infection, or influenza or COVID-19) in combination with the microscopic or cultural detection of *Aspergillus* spp. in a sample of the lower respiratory tract and/or a GM index of >0.5 in serum and/or >0.8 in bronchoalveolar lavage (BAL).

Several laboratory tests for diagnosing IPA are commercially available, e.g., fungal stains, culturing, biomarkers and molecular methods [7]. For any of these test methods, different providers use distinct techniques. These differ regarding their statistical/analytical values to diagnose IPA. In the last couple of years, new biomarkers, as well as the use of new targets, have been described for the detection of invasive aspergillosis (IA) in critically ill or high-risk patients, contributing to a more reliable diagnosis of IPA.

There are several studies investigating the clinical and diagnostic trials to detect an IPA in the patient cohort of hematological patients [3,7,9,10]. Additionally, fewer studies investigate clinical signs and diagnostic methods in the ICU patient cohort. For an appropriate therapy and thus a positive patient outcome, it is crucial that a reliable diagnosis of IPA, even in the early stages, is performed.

**Figure 1:**
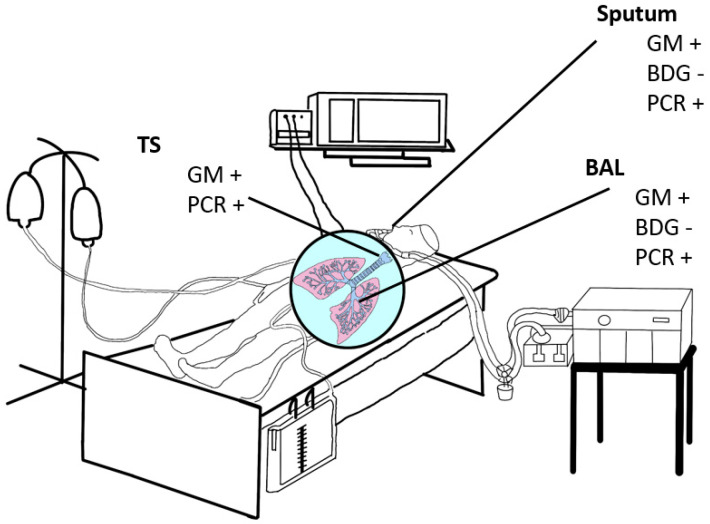
Schematic illustration of specimen used for diagnosis of IPA. BAL-bronchoalveolar lavage; BDG—(1→3)-β-d-glucan 1,3; GM-galactomannan; TS-Tracheal secretion; +—recommended; −—not recommended.

This review provides a summary of microbiological, non-culture-based diagnostic tools for the detection of IPAs in different (respiratory) specimens (Figure 1) from ICU patients, including COVID-19-associated pulmonary aspergillosis (CAPA).

## 2. Non-Culture-Based Diagnostic Tools to Detect an IPA

### 2.1. (1→3)-β-d-Glucan

Non-culture-based methods, e.g., GM and BDG, reduce the time needed to identify IPA in comparison to culture-based methods. Whereas GM from both serum and BAL samples is recommended as a reliable marker in the early detection of IPA [4,7], EORTC/MSG consensus definitions of invasive fungal diseases (IFD) include BDG as mycological evidence while not recommending it for use in clinical trials or for defining IPA as BDG is not specific for any invasive fungal disease [7]. For other specimens like BAL, data are heterogeneous, and no official recommendations by organizations or consortia exist.

BDG is a major cell wall constituent of most medically important fungal species [11]. During IFD, it is released into blood and tissues. Exceptions are cryptococci, Zygomycetes (such as *Absidia*, *Mucor* and *Rhizopus*) and *Blastomyces dermatitidis* which are known to have little or no BDG and thus, glucan is not detected during infection with these organisms [11,12,13].

In a meta-analysis from 2016, Shi et al. reported a much lower sensitivity and specificity of BAL BDG measurement than those of serum BDG (52% and 58%, respectively) with the limitation of high heterogeneity [14]. Study cohorts included patients with and without immunosuppression as well as from ICUs and general wards. Even after the exclusion of the outlier study and reappraisal, performance characteristics were still poor (45% sensitivity and 62% specificity) [14].

While serum BDG has a very high negative predictive value (NPV) in IFD [15], false positive results may occur due to iatrogenic contamination, impaired hepatic function and *Nocardia* spp. infections, among others [16]. Weinbergerova et al. reported that the absolute number of neutrophils led to a decreased predictive value of BDG for IPA (*p* = 0.099). Additionally, the correct prediction of IPA with the help of BAL BDG was shown to be reduced in cases with higher BAL volume (*p* = 0.085) [17]. BAL BDG predictive value is not significantly affected in patients with positive bacterial culture from BAL, but colonization with *Candida* might lead to false reactivity of the BAL BDG assay. In addition, repeated testing demonstrated poor reproducibility of the BDG assay in BAL [18]. Therefore, standardization of BAL sampling is crucial but difficult to implement.

In their multicenter prospective study including solely ICU patients (*n* = 44) with hematological and non-hematological underlying disease, Boch et al. analyzed concurrent serum and BAL samples once by conventional culture, GM, BDG as well as *Aspergillus*-specific PCR. BDG showed a sensitivity of 89% but poor specificity of 31% from BAL and even worse specificity from serum (26%) [19]. However, NPV of BAL BDG was the highest of all diagnostic tests performed in this study (91.7%, [61.5–99.8]) and the combined use of GM and BDG from BAL resulted in similar performance values (sensitivity 88.9% [51.8–99.7], specificity 31.4% [16.9–49.3], positive predictive value (PPV) 25.0% [11.5–43.4] and NPV 91.7% [61.5–99.8]).

Due to different manufacturers, assays have different methods for BDG detection and cut-off values [20]. Comparisons between different assays have mostly been performed between Fungitell (Associated Cape-Cod, Inc., East Falmouth, MA, USA) and Wako (Wako Pure Chemical Industries, Tokyo, Japan) assays using serum but not BAL. Additionally, for an accurate interpretation of Fungitell assay results from serum, the manufacturer recommends testing two to three times per week, which is unlikely to be implemented for BAL sampling. Therefore, single BAL testing will be the most likely modus operandi and, therefore, may impact sensitivity and PPV [21]. Modifying the BDG assay cut-off from 80 pg/mL to 200 pg/mL, as shown by Prattes et al., did not substantially increase specificity [22].

Despite a good NPV, the diagnostic value of BAL BDG seems to be low, whereas studies focusing on ICU settings are scarce. Therefore, many ambiguities exist regarding its diagnostic utility as a stand-alone test or in combination with other assays and whether multiple BDG measurements per week from BAL are necessary for accurate interpretation, among others. Currently, there are no clinical studies examining the utility of BAL BDG in children or for therapeutic response monitoring.

### 2.2. Galactomannan

There is plenty of data on the use of distinct GM assays in BAL samples for the identification of IPA. Three different test procedures for measurement of GM are currently available. The classic GM assay is an enzyme-linked immunosorbent assay (EIA), a chemiluminescence immunoassay (CLIA) and the lateral flow assay (LFA). The GM assays are available from Platelia (Bio-Rad Laboratories, Marnes-la-Coquette, France) and Euroimmun AG (Lübeck, Germany), the CLIA from Vircell S.L. Parque Tecnológico de la Salud (Granada, Spain) and the LFA from OLM Diagnostics (Newcastle Upon Tyne, UK) and IMMY (Oklahoma, OK, USA).

#### 2.2.1. Testing of Sputum Samples

Among others, the detection of GM in sputum samples has a special indication from patients with cystic fibrosis. In this context, the determination of GM serves along with other test results (e.g., total IgE, anti-*A. fumigatus* IgG and IgE, PCR) in a classification of *Aspergillus* disease (bronchitis, colonization, sensitization) [23]. There is only one study in which GM was investigated in the specimen “sputum” of hematological patients. With a cut-off index of 1.2, sensitivity was 100% compared to 66.7% in BAL and 83.3% in serum samples. The specificity was 62.2% [24]. In a few studies, the role of GM detection in sputum samples of non-immunocompromised patients was investigated. In patients with chronic pulmonary aspergillosis, compared to the clinical diagnosis, sputum GM showed a sensitivity of 77% and a specificity of 71%, compared to the criteria culture and IgG positivity, sensitivity and specificity were 77% and 78%, respectively, when using a cut-off of 0.71 in sputum samples [25].

In patients with COPD, the sputum GM OD index was higher in those with bronchiectasis (index 3.7) than in patients without bronchiectasis (0.7). Sputum GM detection correlated with a positive *Aspergillus* culture and PCR results [26]. In another study, sputum samples of patients with allergic bronchopulmonary aspergillosis (ABPA) (*n* = 33) and chronic pulmonary aspergillosis *n* = 126) were investigated by using culture, PCR and GM. The culture was positive in 13%. Depending on the cut-off index (1.0, 4.5, 6.5), the sensitivity of the GM test was 87%, 67%, and 69%, specificity was 31%, 65% and 80% [27]. In a study using induced sputum samples of 38 immunocompetent patients (74% COPD or bronchiectasis) with proven/probable aspergillosis and 89 patients with no aspergillosis, the diagnostic utility of PCR, culture, GM and LFA was investigated. Sputum GM (OD index >2) and BAL GM (>1.0) had a similar sensitivity (84% and 86%, respectively), but the specificity was lower in sputum samples than in BAL (87% vs. 94%). Similarly, the LFA had a comparable specificity of 91% but a lower sensitivity (63%) in sputum than BAL GM [28]. Taken together, the analysis of sputum samples GM detection might be helpful in the diagnosis of aspergillosis in non-immunocompromised patients in which BAL sampling is not possible. By now, no study compares the tests of different companies for the examination of sputum samples.

#### 2.2.2. Testing of BAL Samples

The role of GM testing in BAL has been extensively studied in hemato-oncological patients. In a meta-analysis, a sensitivity of 85% and a specificity of 86% were found when using a cut-off index of 1.0 [29]. The updated EORTC/MSG consensus definition recommends a cut-off index of 1.0 for GM EIA (Platelia) in BAL samples, also with a sensitivity of 75–86% and a specificity of 94–95%. Sensitivity and specificity were similar whether or not haematological malignancies are the underlying disease (85%/87% sensitivity and 91%/89% specificity). If serum/plasma shows an OD ≥ 0.7, a BAL result of ≥0.8 should be interpreted as a relevant result, also [10].

In a large study including 188 patients (35 with COPD and 153 with immunosuppression), the sensitivity was 77.4% and specificity 96.2% when using an index of ≥1.0 [29]. The sensitivity in COPD patients was lower (66.7%) than in immunosuppressed patients (87.8%), but the specificity was nearly identical (94 to 96%) in both patient groups. In the COPD cohort, sensitivity increased to 88.9% when using a cut-off of ≥0.5, but specificity was reduced to 88.4% [30]. In another study, a cut-off of 1.25 was optimal, with a sensitivity of 90.9% and a specificity of 96.3% in COPD patients [31]. A cut-off of 0.8 was proposed [32].

In influenza-related aspergillosis a proposal for a case definition from 2020 recommended a BAL cut-off of 1.0 [33]. In COVID-19 patients, only restricted use of bronchoscopy has been recommended at the beginning of the pandemic to protect the BAL-sampling staff from the potential risk of infection due to aerosol generation. In this context, other materials were preferred in these patients, for example, tracheal secretion. Also, for this material, a cut-off of ≥1.0 was used [34]. Furthermore, in ventilated patients, microaspiration of GM-containing enteral nutrition might be a disruptive factor. Finally, the disadvantage of tracheal secretion is that this material is frequently mucoid and often needs pretest dilution. Overall, the best cut-off value for ventilated patients is not clear. In a small study with 32 ventilated ICU patients, both BAL and tracheal secretion showed nearly identical sensitivity and specificity with a cutoff of ≥1.0 [35]. The 2020 ECMM/ISHAM consensus criteria for the management of CAPA defined for GM-EIA a single cut-off >1.5 for BALs and a single cut-off >4.5 for non-bronchoscopic BAL samples. In the case of more than one material, cut-offs of >1.2 were classified as positive [4]. For sputum, a value of >4.5 was used [4]. Results of 241 respiratory samples of COVID-patients showed, for both *Aspergillus* PCR and GM-EIA, a sensitivity of 90% and a specificity of 77% [36] when using the cut-offs described by Koehler et al. [4].

In a study that compared tracheal secretion to BAL, a cut-off of ≥1 was used for BAL and ≥2 for tracheal secretions, resulting in 75% sensitivity and 81.2% specificity [37]. In an earlier study, the same group defined an index of 2.0 with a sensitivity of 57.1% and a specificity of 81.5% for the GM-EIA and 60% sensitivity and 72.6% specificity for the GM lateral flow assay [38].

The GM-EIA and GM-LFA for BAL samples were analyzed in a study of CAPA [39]. All studied samples were classified as probable CAPA. GM-LFA in BAL showed similar diagnostic capabilities to the classic GM assay, but it is faster for diagnosing CAPA. GM-LFA showed a sensitivity of 60.6%, specificity of 88.9%, PPV of 71.4% and NPV of 83.1% when compared with BAL culture, respectively. GM-EIA showed a sensitivity of 54.5%, specificity of 91.7%, PPV of 75%, and NPV of 81.5% for BAL samples, respectively. Results show that the GM-LFA can be used as an alternative approach in the absence of GM-EIA testing [39].

In a multi-center study, BAL GM and GM-LFA were compared for the diagnosis of IPA [40]. In total, 296 patients with various diseases (65% without an underlying hematological malignancy) were included in the study. The cases were classified as proven (*n* = 2), probable (*n* = 56), putative (*n* = 30), possible (*n* = 45), and no IA (*n* = 162). LFA assay from BAL samples demonstrated reliable diagnostic performance for IPA, and the authors suggested it may be used as a rapid test where GM testing is not quickly available. LFA could differentiate between probable/putative or proven IA versus no IA with an area under the curve (AUC) of 0.865 (95% CI 0.815–0.916), including 88 with IPA versus 162 without IPA. When an ODI cut-off of 1.5 was used, a sensitivity/specificity of 74%/83% was recorded. A sensitivity of 82% but lower specificity of 73% were obtained when an ODI cut-off of 1.0 was used [40].

The diagnostic capabilities of two LFA methods (Olm and IMMY) were compared in a study by Scharmann et al. [41]. Two hundred BAL samples were analyzed, including 24 patients with hematological malignancy (12%), 22 solid tumor patients (11%), 34 solid organ transplantation patients (17%), 18 patients with autoimmune diseases (9%), 41 lung disease patients (20.5%), 18 patients with a cardiological disease (9%), 39 patients with other diseases (19.5%) and four patients where no data were available (2%). After testing, none of the samples could be defined as proven IPA. The total agreement between the two assays was 84%, with the LFA (IMMY) having a sensitivity/specificity of 88.9% and 55.1% and the lateral flow device (LFD, Olm) having a sensitivity/specificity of 93.3% and 46.1% according to Blot criteria. Authors conclude that a negative LFA result can be used to rule out an IPA in a heterogeneous group of ICU patients characterized by Blot criteria [41].

In a study from 2008, Meersseman et al. investigated the role of GM in BAL and serum. In total, 110 patients from the ICU (hematologic malignancy, cancer, solid organ transplant, steroid use, immunosuppressive treatment, Child C cirrhosis, HIV) were included in this study. Twenty-six of the patients had proven IA. The sensitivity and specificity of GM detection in BAL fluid were 88% and 87%, respectively, while sensitivity in serum was only 47%. In total, 11 of the 26 proven cases remained negative in BAL culture and serum GM, while BAL-GM found them all positive. The authors discuss the usefulness of BAL-GM testing for the exclusion of IA in the ICU [42].

There is a significant difference between the sensitivities/specificities calculated in the different studies. This can be explained by the fact that some authors used an adapted cut-off value while others did not. There are certain conclusions that could be drawn from the above-mentioned studies: Firstly, BAL samples seem to be more reliable than serum or blood in GM assays [39,42,43]. Secondly, the BAL-GM assay seems to have the overall highest specificity/sensitivity; however, a clinical standard is lacking. Therefore, the efficiency of assays such as the LFA is currently being investigated, with a good diagnostic performance already being proven in some studies.

### 2.3. Aspergillus PCR Assay in BAL

The PCR assay is one of the most commonplace detection methods in clinical settings. Several publications from the past years have discussed the performance of the PCR assay in BAL as a diagnostic tool for IPA in different patient cohorts. In a review of commercially available PCR tests, sensitivities of 68–94% and specificities of 80–98% were found in different patient groups [44].

Scharmann et al. evaluated three PCR assays for the detection of IPA in immunocompromised patients [45]. In this study, the variation between different manufacturers becomes clear. Here, statistical analysis revealed a variation in the sensitivity from 60.0% to 80.0%, the specificity from 73.2% to 96.7%, the same as the PPV varies between 26.7% and 70.0% and NPV between 95.4% and 96.8% [45].

### 2.4. Combination of Diagnostic Methods

Since the emergence of CAPA and its high mortality rates, the need for an efficient diagnosis of IPA has become evident [46]. Some studies have published results on the combined diagnostic capabilities of the GM and PCR assays in non-hematological patients, which is significantly improved compared to the use of one of these methods solely.

A study of 63 CAPA patients where BAL samples were analyzed assessed the importance of *Aspergillus* species in ventilated patients. Here, assays such as BAL-GM/ serum-GM or BAL-PCR were used. Probable CAPA was diagnosed in 17% of patients, not all of whom had EORTC/MSG host factors for IPA. Sensitivity (range) for PCR, BAL GM and serum GM was 44% (13.7–78.8), 55.6% (21.2–86.3) and 33.3% (7.5–70.1), respectively. Specificity (range) for PCR, BAL GM and serum GM was 94.3% (80.8–99.3), 94.3% (80.8–99.3) and 97.1% (85.1–99.9), respectively. They were able to withhold treatment in three of 15 patients with positive screening (20%) but negative BAL GM results. They conclude that positive culture, molecular detection or antigen detection of *Aspergillus* species do not necessarily indicate infection [47].

A study of the BAL fluid of 101 ICU patients (mostly COVID-19 or immunocompromized non-COVID-19 patients) showed that *Aspergillus*-PCR in BAL could improve the diagnostic accuracy of BAL GM. In the ICU COVID-19 group, 15 of 59 patients were diagnosed with proven CAPA. In the ICU non-COVID-19 cohort, three patients had putative (possible) IPA, while 24 had no putative IPA. In the immunocompromised group, 13 patients were diagnosed with proven/probable IA, while 12 had no IA. *Aspergillus* PCR sensitivity was 64% (95% CI 47–79) and specificity 99% (95% CI 93–100). *Aspergillus* PCR sensitivity was 40% (95% CI 19–64) in ICU COVID-19, 67% (95% CI 21–93) in non-COVID-19 ICU patients and 92% (95% CI 67–98) in the immunocompromised patients [48].

### 2.5. Novel Biomarker/Targets

Several studies investigating new biomarkers or new targets for the detection of IA in critical-ill or high-risk patients have been published in recent years.

The knowledge of the pathogenesis of IPA identifies the interaction of the ciliated epithelium and innate immune system, including resident alveolar macrophages and dendritic cells, and recruited inflammatory cells, as the first line of defense against inhaled fungal spores [49]. These cells express a large repertoire of immune receptors, sensing pathogen motifs and driving the secretion of cytokines and chemokines that control innate and adaptive immune responses [50].

The question is if a specific inflammatory signature of cytokines and chemokines is specific for IA. In one study, cytokine profiles in BAL samples from patients with IPA were compared to matched control patients [49]. It was shown that a subset of alveolar cytokines could significantly discriminate cases of *Aspergillus* infection from those without infection. Furthermore, it was reported that two distinct clusters of highly correlated cytokines (IL-1β, IL-6, IL-8, IL-17A, IL-23, and TNFα) were differentially expressed between cases of IPA and controls. IL-8 was the best-performing cytokine, with alveolar levels ≥904 pg/mL predicting IPA with elevated sensitivity (90%), specificity (73%), and NPV (88%). This was the first study of its kind, including BAL samples. High serum IL-8 levels were reported as a reliable blood biomarker for IPA [51].

Cytokines also play a major role in ICU patients with COVID-19 and are a risk for developing CAPA. The cytokine storm that occurs in COVID-19, instead of activating a competent immune response to possible opportunistic infections, causes the dysregulation of the immune system. This is linked to a reduction of lymphocyte count, dampening of cell-mediated fungicidal activity and ineffective conidial killing, creating a fertile ground for fungal invasion. The cytokine pattern expressed in severe COVID-19 shares some similarities with severe IPA (i.e., high levels of TNF-α, IL-1, IL-6, IL-8, IL-10, and low levels of IFN-γ) [52]. So far, studies evaluating cytokines in COVID-19 patients as diagnostics markers for IPA do not exist.

Another biomarker initially showing promising results for diagnosing IPA is triacetylfusarinine C (TAFC). TAFC is a fungal-specific molecule that is produced by a limited number of molds, among them *A. fumigatus*. TAFC is a secreted siderophore [53]. The AUC for TAFC differentiating probable/proven from no IPA was 0.601 (0.425–0.777, n.s.) in a study including 44 samples from 15 patients with IPA and 29 controls. Sensitivities of BAL-GM were increased from 53% to 73% (1.0 ODI GM cut-off) and from 73% to 87% (0.5 ODI GM cut-off) when combined with TAFC [54]. Another recent study evaluated existing and novel biomarker tests and reported that TAFC was detectable in only three of 38 proven/probable IPA patients [28], indicating no benefit for IPA diagnosis. Further studies are needed to know if TAFC in BAL is a helpful add-on in the diagnosis of IPA.

TAFC can also be found in urine. The urine TAFC, normalized to creatinine, was measured in high-risk patients [55]. TAFC/creatinine sensitivity, specificity, and positive and negative likelihood ratio for probable versus no IPA (cut-off ≥3) were 0.86, 0.88, 6.86 and 0.16 per patient. This approach shows the advantage of non-invasive sampling. The determination of the siderophore TAFC was performed in most studies by mass spectrometry. Mass spectrometry is not available everywhere and needs expertise and experience. However, it was also shown that TAFC can be rapidly measured by interference-enhanced Raman spectroscopy [56].

In patients with suspected fungal pneumonia, an *Aspergillus* secondary metabolite signature in breath can identify individuals with IPA [57]. In an assessment of volatile *Aspergillus* metabolites in the breath of 64 patients with suspected fungal pneumonia, a secondary metabolite signature of α-trans-bergamotene, β-trans-bergamotene, a β-vatirenene–like sesquiterpene, or trans-geranylacetone identified patients with IPA with 94% sensitivity and 93% specificity. These results seem promising but were not confirmed so far. As a diagnostic platform, gas chromatography with a mass spectrometry approach was used.

Another approach detecting fungal pathogens like *Aspergillus* in respiratory secretions is proteome analysis. Proteome analysis of BAL reveals host and fungal proteins highly expressed during IPA in mice and humans [58]. Overall, 16 fungal proteins were identified that were specifically detected during infection and may be valuable candidates for biomarker evaluation in the future.

Novel diagnostic markers are urgently needed. Clinicians would like to have fast, easy-to-perform diagnostic tests with high sensitivity and specificity and low costs. All discussed new markers cannot fulfill these criteria. Another limitation is that the described methods require, in most cases, a high degree of expertise.

## 3. Conclusions

Taking everything into account, it is crucial to detect an IPA reliably in ICU patients. Based on recommendations from EORTC/MSG criteria, Blot et al., Bassetti et al., and Schauwvlieghe et al., not only host factors and clinical signs should be fulfilled, but also microbiological diagnostic is an important milestone for IPA diagnostics [7,9,59]. Various studies investigated the diagnostic tools with different results, which led to the observation of non-homogeneous patient groups. A comparison of the different studies is critical due to the different cohorts observed. Here, we pointed out the most reliable microbiological non-culture-based methods to detect an IPA in ICU patients. The combination of different methods and the investigation from different specimens (BAL and serum samples) seems to bring the most reliable results. BDG in serum shows a high NPV, while GM EIA from BAL samples showed the highest specificity for ICU patients. The cut-off, which is well established in hemato-oncological patients (index ≥1.0), is not so clear in other patient groups. The cut-off may differ in different patient groups in the future. The GM LFA showed a lower specificity than the EIA, but at the same time, it is faster and more easy to handle. Detecting *Aspergillus* with a commercially available PCR, especially the AsperGenius, which has been shown to be the best-evaluated method, showed high specificity and NPV in all patient groups. So far, new biomarkers or targets (e.g., cytokines, TAFC or secondary metabolite) are not clinically investigated sufficiently, making evaluation of these biomarkers necessary in the future.

## Data Availability

All references can be found in PubMed^®^ the National Library of Medicine.
